# Deficiency of the NAD(P)HX metabolic repair system: a treatable mitochondrial disease

**DOI:** 10.1186/s13023-026-04218-4

**Published:** 2026-01-23

**Authors:** Chaolong Xu, Hong Jin, Jiuwei Li, Zhimei Liu, Weihua Zhang, Ji Zhou, Ruoyu Duan, Yang Liu, Minhan Song, Zixuan Zhang, Tongyue Li, Danmin Shen, Ying Zou, Junling Wang, Hua Li, Huafang Jiang, Fang Fang

**Affiliations:** 1 Department of Neurology, Beijing Children’s Hospital, Capital Medical University, National Center for Children’s Health, Beijing, China; 2 Laboratory for Clinical Medicine, Capital Medical University, Beijing, China; 3 Department of Pediatrics, The Third Affiliated Hospital of Zhengzhou University, Zhengzhou, China; 4 Department of Pediatrics, WeiFang Maternal and Child Health Hospital, Weifang, China

**Keywords:** NAD(P)HX dehydratase, NAD(P)HX epimerase, Nicotinamide nucleotide repair system, Nicotinamide, Treatment

## Abstract

**Objective:**

This study aims to explore the clinical characteristics of patients with NAD(P)HX metabolic deficiency and their prognosis after nicotinamide treatment.

**Methods:**

This study retrospectively analyzed the clinical characteristics, efficacy of nicotinamide treatment, and prognosis of patients with genetically confirmed NAD(P)HX metabolic defects admitted to Beijing Children’s Hospital from January 2016 to January 2025, as well as cases previously reported in the literature. The log-rank test was used for survival analysis, and the prognosis was evaluated using the Modified Rankin Scale (mRS).

**Results:**

Nine patients were analyzed, including eight with NAXE deficiency and one with NAXD deficiency, seven of whom received nicotinamide treatment (180–500 mg/day). With a median follow-up of 3.92 years [range: 0.50–6 years, interquartile range (IQR) = 2.42 years], the overall prognosis was favourable. All seven treated patients survived, three of whom were able to attend school normally, and no significant adverse reactions were observed during treatment. Combined with previous studies, a total of 59 patients were included for analysis (14 cases of NAXD deficiency and 45 cases of NAXE deficiency), with an overall mortality rate of 66.7%. Among the 21 patients who received niacin/nicotinamide treatment, 17 survived (80.95%), whereas only two untreated patients survived, and 85.45% of the untreated patients died within 2 years of onset. Respiratory failure was the most common cause of death.

**Conclusions:**

NAD(P)HX metabolic defects are rare mitochondrial diseases with high mortality and morbidity rates. Early identification and timely initiation of nicotinamide treatment are crucial for improving patient prognosis and quality of life.

**Supplementary Information:**

The online version contains supplementary material available at 10.1186/s13023-026-04218-4.

## Background

A variety of highly specific and highly conserved metabolic enzymes are key elements for maintaining the normal functions of human cells. Although these enzymatic reaction pathways play a central role in vital activities, even under normal physiological conditions, the metabolic processes may still produce potentially toxic metabolites. Fortunately, “metabolite repair” enzymes promptly activate protective mechanisms—through catalytic conversion reactions, they transform these harmful substances into non-toxic and chemically stable compounds, thus significantly reducing their potential threats to the body [1].

**Fig. 1:**
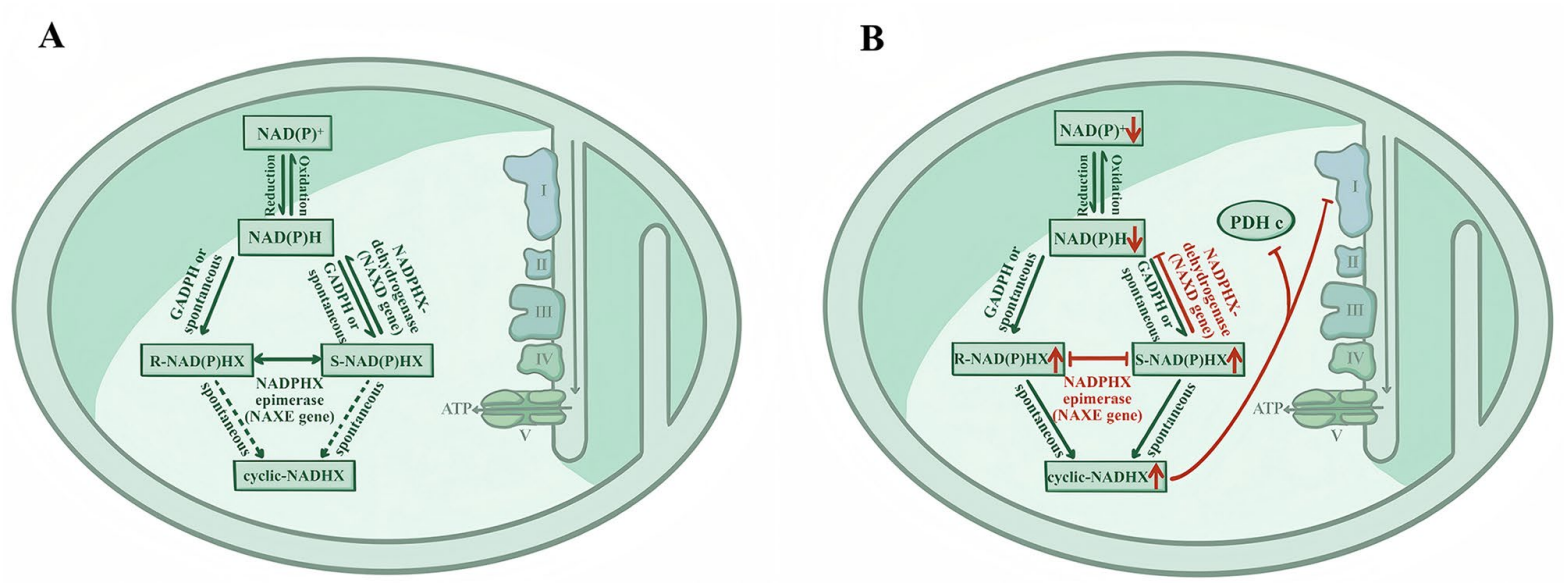
Schematic overview of NAD(P)^+^/NAD(P)H metabolism and the NAD(P)HX repair system. (**A**) NAD(P)HX repair system at metabolic equilibrium under normal/healthy conditions. (**B**) The potential pathological mechanism of NAXE/NAXD deficiency is characterized by a significant increase in cyclic-NADHX production, which can inhibit the activity of cellular Complex I and PDHc and trigger metabolic imbalance in the NAD^+^/NADH coenzyme system. GADPH: Glyceraldehyde-3-phosphate dehydrogenase; Complex I: Mitochondrial Complex I; PDHc: pyruvate dehydrogenase

The mitochondrial nicotinamide adenine dinucleotide (phosphate), hydrated form [NAD(P)HX] metabolic repair system, a highly conserved protective mechanism essential for intracellular repair, comprises two key enzymes: NAD(P)HX isomerase (NAXE, OMIM #608862) and NAD(P)HX dehydrogenase (NAXD, OMIM #615910). Nicotinamide adenine dinucleotide (NADH/NAD^+^) and nicotinamide adenine dinucleotide phosphate (NADPH/NADP^+^), two core redox coenzymes in organisms, are not only the core donors driving electron transfer in the mitochondrial respiratory chain, but also the necessary molecular basis for the normal operation of numerous metabolic pathways [2]. However, the reduced form of NAD(P)H is unstable. It can spontaneously undergo hydration in a weak acidic environment or at high temperatures and can undergo hydration as a side reaction of glyceraldehyde 3-phosphate dehydrogenase (GAPDH), forming a toxic derivative, NAD(P)HX [3, 4]. Under normal physiological conditions, the body converts the abnormal metabolite NAD(P)HX into the normal cofactor NAD(P)H through the action of the NAXE and NAXD enzymes, maintaining cellular homeostasis (Fig. 1A). When mutations occur in NAXE or NAXD genes, the abnormal metabolite NAD(P)HX cannot be efficiently cleared and accumulates within cells. This accumulation not only inhibits the activities of Mitochondrial Complex I and pyruvate dehydrogenase, but also reduces the levels of endogenous normal NAD(P)H, leading to the disruption of cellular metabolic networks and mitochondrial dysfunction, inducing related diseases (Fig. 1B) [5, 6].

Mutations in NAXE and NAXD genes lead to mitochondrial nicotinamide adenine dinucleotide (NAD(P)HX) metabolic repair deficiencies, which are fatal neurodegenerative diseases in infants and young children [7, 8]. These diseases are known as Progressive Encephalopathy with Brain Edema and/or Leukoencephalopathy-1 (PEBEL1; OMIM #617186) and PEBEL2 (OMIM #618321). Clinically, these diseases are characterized by the rapid onset of acute deterioration of neurological function triggered by fever or trauma, specifically manifesting as motor disorders, dystonia, ataxia, epilepsy, metabolic encephalopathy, and acute respiratory failure, which may or may not be accompanied by characteristic severe skin lesions. These symptoms can be aggravated by fever and gradually progress to brain atrophy and dementia within 1 to 2 years after onset, ultimately leading to the premature death of patients [7–10].

Nicotinamide, also known as vitamin B_3_, is a precursor for the synthesis of NAD^+^. The NAD^+^ pool in skin fibroblasts of patients with NAXD mutations and yeast is significantly reduced, and exogenous supplementation with nicotinamide can restore the level of NAD^+^ in the body [11, 12]. In 2019, we reported a clinical case of a patient with NAXD deficiency who was successfully treated with nicotinamide and achieved long-term survival [13]. In contrast, in six similar cases worldwide, none of the patients received nicotinamide therapy, and all died before the age of 5 [7]. Building on our prior treatment experience with NAXD deficiency, our center successfully treated six patients with NAXE deficiency and conducted long-term follow-up with an overall favourable prognosis.

Currently, multiple research centers worldwide have cumulatively reported 14 cases of NAD(P)HX metabolic deficiency patients treated with niacin/nicotinamide [12, 14–24]; the overall survival rate was 71.4% (10/14). Given that the existing reports are case studies from different centers, there are differences in treatment dose selection and intervention timing, and patients possibly exhibit varying responses to treatment owing to racial and genetic background disparities. Here, we aimed to systematically analyse the clinical characteristics, treatment protocols, and long-term prognosis of nine patients with NAD(P)HX metabolic deficiency diagnosed and treated in our center. Additionally, we conducted a systematic review to deepen the understanding of such diseases, promote early diagnosis and standardized treatment, and improve patient outcomes.

## Methods

### Participants

This single-center retrospective study enrolled patients identified from the Chinese Mitochondrial Disease Network Registry (MitoC-NET) between January 2016 and January 2025. All patients were confirmed by genetic testing to carry NAXE or NAXD genes mutations, and all detected variants were likely pathogenic or pathogenic. Written informed consent was obtained from all patients or their guardians before inclusion in the study. This study was approved by the Ethics Committee of the Beijing Children’s Hospital, Capital Medical University ([2022]-E-121-Y), and all procedures were conducted in accordance with the principles outlined in the Declaration of Helsinki.

### Genetic analyses and pathogenicity assessment

Standard procedures were used to perform blood mitochondrial DNA (mtDNA) sequencing for each patient, and whole-exome sequencing was performed for the patient and their parents. The experimental workflow was as follows. The coding regions of the whole exome were enriched using the Agilent SureSelect Human All Exon V6 Kit. Subsequently, 150-bp paired-end sequencing was conducted on an Illumina HiSeq2000 platform. The generated sequencing reads were precisely mapped to the human reference genome (UCSC Genome Browser build hg19) using the Burrows–Wheeler Aligner 20 (BWA) [25]. The interpretation of variants was conducted in strict accordance with professional guidelines formulated by the American College of Medical Genetics and Genomics guidelines, and standardized annotation was carried out using the nomenclature system of the Human Genome Variation Society (HGVS) [26, 27]. To ensure the validity and accuracy of our findings, the identified mutation sites were rigorously validated using Sanger sequencing.

### Measurement of the NADH/NAD^+^ ratio in human fibroblasts

To investigate whether there was an imbalance in the NADH/NAD^+^ ratio during NAXD gene deficiency and to provide a theoretical basis for nicotinamide treatment, we used the NADH/NAD^+^ Glo Assay Kit (Cat. No. G9072, Promega) to measure the NADH/NAD^+^ ratio in the skin fibroblasts of Patient 9 (Pt-9). The specific detection protocol was as follows. For NAD^+^ measurement, 25 µl of 0.4 N HCl was first added to the supernatant and mixed thoroughly, followed by incubation at 60 °C for 15 min to degrade NADH. After incubation, the samples were equilibrated at 20 °C for 10 min, followed by neutralization and quenching: 25 µl of 0.5 M Tris solution was added to NAD^+^ samples, and 50 µl of HCl/Tris mixture was added to NADH samples. Finally, the samples were mixed with the detection reagent in 1:1 volume ratio, incubated at 20 °C for 30 min, and the luminescence signal was detected.

### Data collection

We systematically collected clinical, metabolic, genetic, neuroradiological, and treatment-related data using standardized forms, covering both the acute phase and remission phase of the patients. Medical history encompassed sex, age at disease onset, disease duration, initial clinical symptoms, as well as neurological and non-neurological manifestations. Phenotypic data were standardized in accordance with Human Phenotype Ontology terms. In-depth clinical evaluations were conducted, and auxiliary examination results were obtained, including serum/cerebrospinal fluid lactate levels, complete blood count, biochemical tests, electrocardiography (ECG), echocardiography, cranial magnetic resonance imaging (MRI), and genetic variation information.

### Treatment and follow-up

Once diagnosed with NAXD or NAXE defects, nicotinamide therapy was initiated immediately in all patients. The treatment dosage was based on Pellagra [28], with an initial dose of 100 mg/day, which was gradually titrated to 500 mg/day at a rate of 100 mg weekly, based on individual tolerance and therapeutic response. In cases where patients developed adverse reactions, such as flushing or perioral rash, the dose was reduced stepwise to a tolerable level. Given the high clinical suspicion of mitochondrial disease in 5 patients (Pt-2, Pt-4, Pt-5, Pt-7, and Pt-9), short-term combined vitamin therapy was added alongside nicotinamide treatment in accordance with the standard therapeutic regimen for mitochondrial disease during the acute encephalopathy phase, specifically including vitamin B_1_ (10–40 mg/kg/day), vitamin B_2_ (10–20 mg/kg/day), vitamin E (0.7–1.4 mg/kg/day), coenzyme Q10 (5–30 mg/kg/day), and levocarnitine (50–100 mg/kg/day) [29]. These patients discontinued the combined vitamin regimen immediately after genetic confirmation or resolution of acute-phase symptoms, and maintained long-term monotherapy with nicotinamide alone.

Follow-up started from the date of each patient’s first visit and ended with the last clinical assessment before the article was submitted for publication. Our centre has established a standardised follow-up system for patients with MD: the first follow-up is scheduled 3 months after diagnosis, and the follow-up is conducted every 6 months during the stable phase. The follow-up included standardised assessment items, covering growth and development indicators such as height and weight, functional development status of motor/cognitive/language abilities, and records of seizure frequency; laboratory tests included monitoring cardiac and liver functions, audiovisual function assessment, and magnetic resonance imaging (MRI) review every 6–12 months. At the final follow-up, the Modified Rankin Scale (mRS) was used to evaluate motor function.

### Literature review

A literature search was conducted using Medical Subject Headings such as “NAXE,” “NAXD,” “NAD(P)HX,” “PEBEL1,” “PEBEL2,” “Vitamin B_3_,” “Nicotinamide,” and “Niacin” across the Wanfang, Chinese National Knowledge Infrastructure, PubMed, and Embase databases up to May 2025. All articles were screened, and 28 articles describing cases were included in our review [7–10, 12–24, 30–40]. We extracted the clinical manifestations, laboratory test results, neuroimaging features, genetic information, treatment interventions, and follow-up data for each case according to the reports in the articles.

### Statistical analysis

The means, standard deviations, medians, and interquartile range (IQRs) were calculated. For categorical variables, the chi-squared test or Fisher’s exact test was used for analysis. Univariate and multivariate Cox proportional hazards models were used to calculate hazard ratios (HRs) and 95% confidence intervals (CIs) for assessing prognostic risk factors. Survival analysis was performed using the log-rank test. A post-hoc power analysis was conducted using G*Power 3.1 software to determine the association between nicotinamide therapy and mortality in the study sample.

All other statistical analyses were conducted using SPSS version 25.0 (SPSS Inc., Chicago, IL), with statistical significance defined as *P* < 0.05.

## Results

### Demographic features

Between January 2016 and January 2025, 318 cases of mitochondrial diseases with nuclear gene variants were registered at our centre within the Chinese MitoC-NET registry. Among these, nine patients (2.83%, 9/318) from Han Chinese families were diagnosed with NAD(P)HX metabolic repair system defects, including one case with an NAXD gene variant and eight cases with NAXE gene variants. This group, comprising six boys and three girls, had a median age at onset of 2.75 years (range: 0.83–3.17 years, IQR = 2.08 years), a median age at diagnosis of 4.33 years (range: 2.58–5.50 years, IQR = 3.33 years), a median time from symptom onset to diagnosis of 0.92 years (range: 0.17–3.5 years, IQR = 0.58 years), and a median age at the last follow-up of 6.00 years (range: 2.83–10.50 years, IQR = 4.08 years).

### Clinical features

**Fig. 2:**
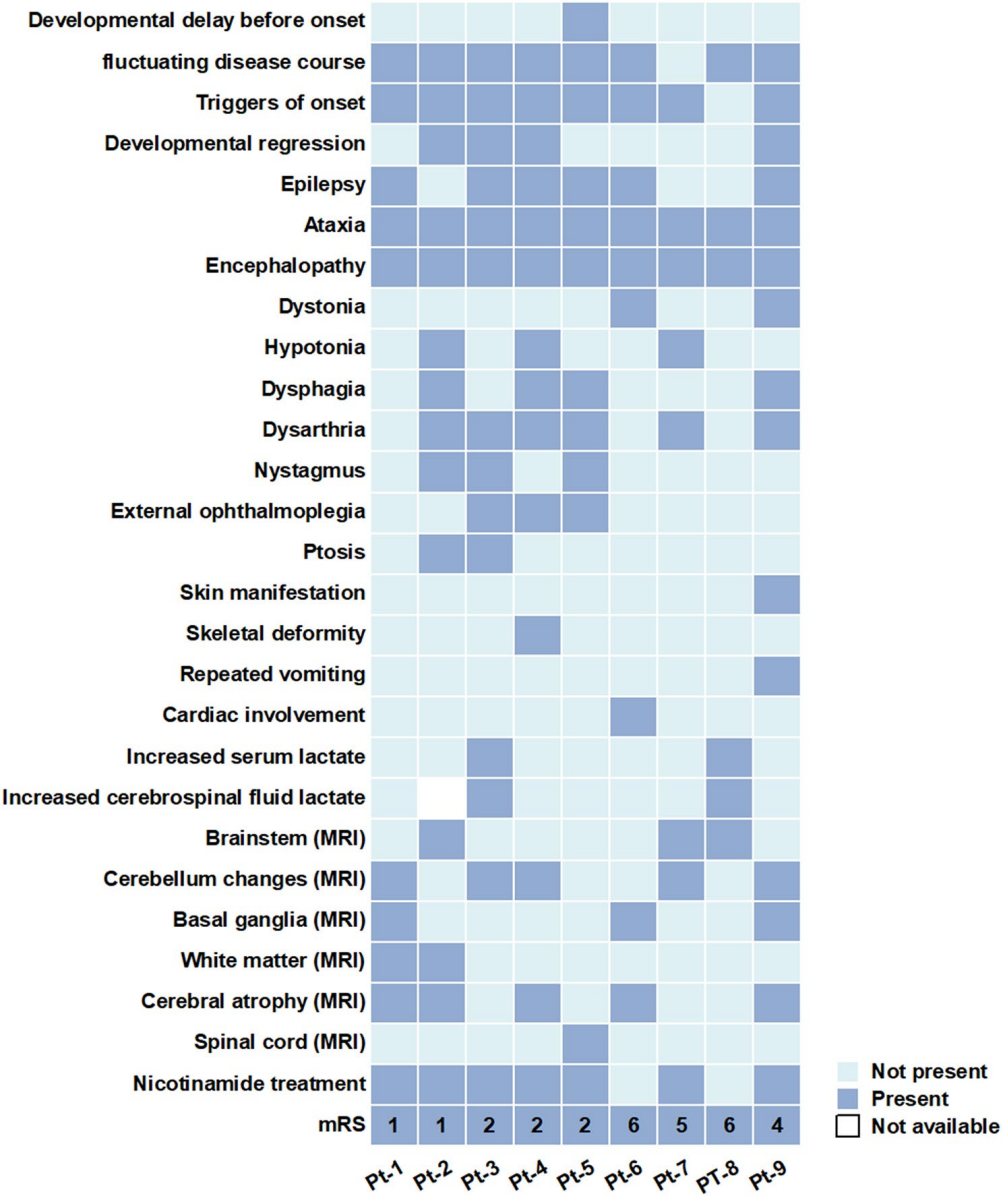
Summarizes the main clinical features, laboratory test, neuroimaging lesion, nicotinamide treatment, and mRS scores at the last follow-up of 9 patients with NAD(P)HX metabolic deficiency. mRS, Modified Rankin Scale score

Eight of the nine patients had normal development before the onset of the initial symptoms, and fever was identified as a triggering factor in eight patients. The predominant onset symptom was ataxia (7/9), followed by epilepsy and muscle weakness, each presenting in two cases. In addition, there was one patient in whom the initial symptom was involuntary movement. Following the onset, all patients developed encephalopathy (disturbance of consciousness and involuntary movement) during the acute phase. Other common symptoms included ataxia (*N* = 9), epilepsy (*N* = 6), dysarthria (*N* = 6), dysphagia (*N* = 4), nystagmus (*N* = 3), external ophthalmoplegia (*N* = 3), dystonia (*N* = 2), and ptosis (*N* = 2). In addition to these neurological manifestations, thoracic deformity (Pt-4), elevated cardiac enzyme levels (Pt-6), and repeated vomiting (Pt-9) were observed in one patient each. Rash was present in only one patient with NAXD deficiency (Pt-9), whereas the remaining eight patients with NAXE deficiency showed no rash. The early disease course showed a remarkably fluctuating nature, with 90% of the patients (8/9) experiencing alternating periods of remission and exacerbation. Some patients (Pt-1, Pt-2, Pt-6, and Pt-9) recovered to baseline levels. The main clinical information of the 9 patients is summarized in Fig. 2.

**Fig. 3:**
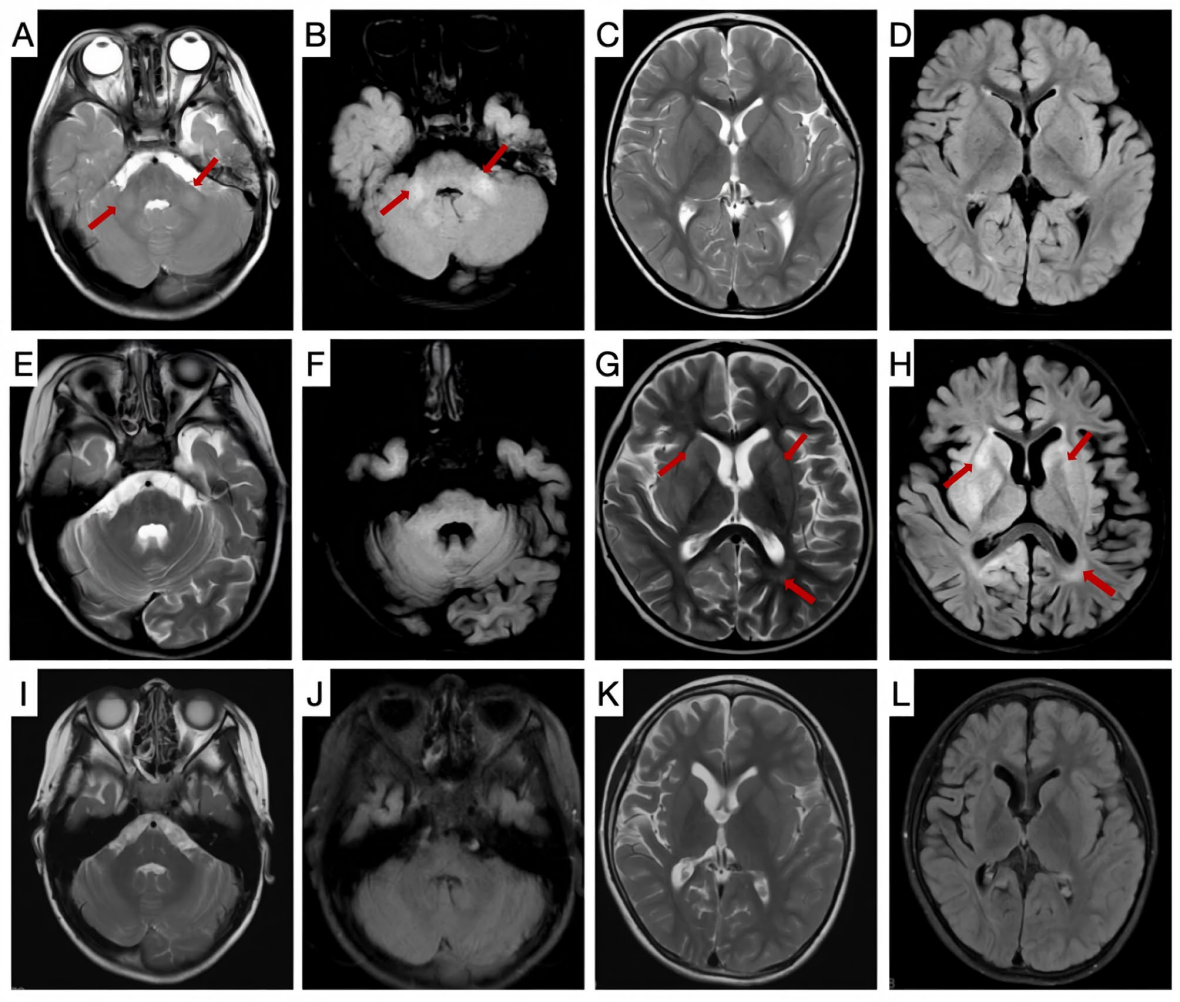
Cranial MRI images of patients with NAXE deficiency. Patient 1 was a 7-year-old girl with a homozygous variant in the NAXE gene (c.733 A > C), who first presented with gait instability at 2 years and 7 months of age. Cranial MRI revealed abnormal signals in the bilateral cerebellar dentate nuclei on T2 and T2 FLAIR sequences (**A**-**D**). After treatment with hormones combined with intravenous immunoglobulin, her symptoms resolved to baseline. Two months later, the patient developed recurrent gait instability and altered consciousness following fever. Repeat MRI (T2 and T2 FLAIR sequences) showed new abnormal signals in the bilateral basal ganglia and periventricular white matter of the posterior horn of the lateral ventricles (**E**-**H**). Nicotinamide therapy was initiated at 5 months of disease onset, and symptoms gradually improved. A follow-up cranial MRI 3 years and 4 months after treatment showed significant improvement in abnormal signals in the cerebellar dentate nuclei, basal ganglia, and periventricular white matter of the posterior horn of the lateral ventricles, but left brain atrophy predominantly in the right cerebral hemisphere (**I**-**L**)

Serum and cerebrospinal fluid (CSF) lactate levels were elevated in two of the eight patients (Pt-3 and Pt-8). Serum lactate fluctuated between 3.71 and 4.54 mmol/L (reference range: 0.5–2.2 mmol/L), and CSF lactate fluctuated between 2.87 and 4.12 mmol/L (reference range: 1.00–2.78 mmol/L). Additional CSF analyses revealed elevated protein levels in both Pt-3 and Pt-8, ranging from 473 to 525 mg/L (reference range: 20–450 mg/L); Pt-3 also exhibited increased CSF pressure. CSF cell counts and autoantibodies associated with autoimmune encephalitis were within the normal ranges. At the early stage of disease onset, only two of the nine patients showed abnormal findings on cranial MRI, with the affected areas being the cerebellum (Pt-1) and brainstem (Pt-7). As the disease progressed, subsequent MRI follow-ups within 1–6 months revealed diverse imaging abnormalities in all patients. The most involved sites were the cerebellum (four cases) and brainstem (three cases), followed by the basal ganglia (two cases) and periventricular white matter (two cases). In addition, one patient had spinal cord lesions, and five patients developed cerebral atrophy changes at a disease duration of 0.25 to 1.00 years (Fig. 3).

### Genetic findings

In eight patients from different families carrying NAXE (RefSeq NM_144772.2) variants, 16 genetic variants were identified, including 13 missense variants, two frameshift variants, and one splice variant. Among these, four novel variants, c.335T > C, c.538_541del, c.402 + 3_402 + 6del, and c.538dup, were previously unreported. Detailed genetic information is provided in Supplementary Table S1. The most clinically significant variant, c.733 A > C (p.Lys245Gln), was identified in 87.5% (7/8) of independent genetic lineages. Five patients (Pt-3, Pt-4, Pt-6, Pt-7 and Pt-8) harboured homozygous genotypes for this variant, and none of their parents had consanguineous relationships. The NAXD gene, which has been previously reported, is not addressed here [13].

### NADH/NAD^+^ ratio in fibroblasts from patients with NAXD deficiency

To investigate the impact of NAXD deficiency on cellular metabolism, we measured the NADH/NAD^+^ ratio in skin fibroblasts from NAXD-deficient patients and their healthy parents under conditions of 37 °C (normal culture temperature) and 42 °C (heat stress). The results showed that the NADH/NAD^+^ ratio in the patient group was significantly higher compared to the control group (fibroblasts from healthy parents), with this difference being more pronounced under heat stress at 42 °C. All observed differences were statistically significant (Supplementary Fig. S1).

### Treatment and follow-up of patients with NAXE deficiency

Among the eight patients in this study, six with NAXE mutations received nicotinamide treatment. Nicotinamide therapy was initiated 0.08 to 3.50 years after disease onset. Except for Pt-4, all patients successfully escalated the dosage from an initial 100 mg/day to 500 mg/day in a stepwise manner without any adverse reactions and with good tolerance. Pt-4 developed flushing and a perioral rash when the dose reached 300 mg/day, which completely subsided after the dose was adjusted to 180 mg/day. After a median follow-up of 3.76 years (range: 0.50–5.08 years) for efficacy and safety, nicotinamide therapy demonstrated remarkable overall effectiveness. No disease fluctuations occurred in any patient during the treatment period, even in the presence of febrile stress. Five patients showed significant relief from ataxia or muscle tone abnormalities 3–6 months after treatment initiation. One year after treatment, sustained improvements were observed in multiple indicators of neurological function, including cognitive function, language expression, and fine motor skills.

Motor function impairment was the primary clinical manifestation of neurological symptoms. During the last follow-up, researchers used the modified Rankin scale (mRS) to conduct a functional assessment of the six treated patients. The median score was 2 points, and five patients had a score of 2 points or below. Three of the six patients (Pt-1, Pt-2, and Pt-4) had been able to regularly attend school at the same age as their peers. Among them, the patient with the NAXE mutation with the most severe condition (Pt-7) initially developed acute ataxia after infection, followed by rapid deterioration of the condition and progression to respiratory failure. Although nicotinamide treatment (500 mg/day) was initiated 1 month after disease onset, the patient remained unconscious and still required continuous respiratory support to sustain life. Regrettably, the other two NAXE-mutated patients (Pt-6 and Pt-8) did not receive nicotinamide treatment because their genetic diagnosis was only confirmed through retrospective analysis; they failed to undergo genetic testing for a definitive diagnosis during their lifetime. These two patients died at the ages of 2.83 and 4.08 years, 1.33 and 2 years after disease onset, respectively, both from respiratory failure following an infection.

### Treatment and follow-up of patients with NAXD deficiency

The patient carrying the NAXD mutation, the world’s first case of NAD(P)HX repair deficiency receiving nicotinamide treatment, gradually underwent an increased nicotinamide dose to 500 mg/day starting at 4 years and 6 months of age (the 20th month of the disease course), while gradually discontinuing other vitamin therapies. After 6 years of long-term treatment and follow-up, the patient demonstrated significant improvements in multiple functional indicators. In the second year of treatment, the skin lesions gradually resolved, and severe spastic symptoms and dysphagia were effectively alleviated, allowing independent eating and proficient use of both hands to operate a mobile phone. Despite several febrile illnesses during the treatment period, the patient’s condition remained stable without signs of recurrence. The latest follow-up showed that the 10.5-year-old patient still mobilises with a wheelchair, but can operate games with both hands. The patient’s swallowing function has returned to normal, cognitive ability has been significantly improved, and he can understand and execute instructions. However, speech expression function was not restored, and the latest mRS score was 4 (5 before treatment). Furthermore, when the patient received nicotinamide at a dose of 500 mg/d for 3 years, a rash appeared around the mouth. After the nicotinamide dose was adjusted to 250 mg/day, the rash subsided, and the therapeutic effect was maintained.

### Safety monitoring

Regular monitoring of patient biochemical parameters showed that none of the seven patients developed drug-related injuries to vital organs such as the liver and kidneys.

### Literature review

To date, 51 patients with NAD(P)HX metabolic repair deficiency have been reported, including 37 with NAXE and 14 with NAXD deficiency. After including newly reported cases from our centre and excluding one duplicate case, 59 patients were finally enrolled for the statistical analysis.

### Clinical features of patients with NAD(P)HX deficiency

In terms of the age of onset, patients carrying NAXE mutations and those with NAXD mutations exhibited significant heterogeneity at the time of onset, which can span from childhood to adulthood (Table 1). Furthermore, 81.25% (39/48) of the patients had normal developmental indicators before the onset of the disease, and 81.82% (45/55) had definite predisposing factors, among which fever or infection was the most common. Regarding the onset of symptoms, both mutations show diverse characteristics, with patients carrying NAXD variants mainly presenting with epilepsy, developmental delay/regression, while those carrying NAXE variants more frequently exhibit ataxia and muscle weakness. Almost all patients show acute progressive neurological impairment with typical symptoms including encephalopathy, developmental regression, epilepsy, hypotonia, and ataxia. With regard to other neurological signs, patients with NAXE variants were more prone to nystagmus, external ophthalmoplegia, and ptosis (Table 1).

**Table 1:** Demographic, clinical characteristics, treatment, and prognosis of cases with *NAD(P)HX* deficiency

	*NAXD (* *N* * = 14)*	*NAXE (* *N* * = 45)*	*Total (* *N* * = 59)*
**Age of onset (years)**	1.00 (0.25–32.00)	1.42 (0.00–22.00)	1.33 (0.00–32.00)
**Male**	8/14 (57.14%)	25/45 (55.56%)	33/59 (45.93%)
**Main neurological symptoms**			
Epilepsy	9/14 (64.29%)	20/45 (44.44%)	29/59 (49.15%)
Ataxia*	6/14 (42.86%)	35/45 (77.78%)	41/59 (69.49%)
Encephalopathy	7/14 (50.00%)	30/45 (66.67%)	40/59 (67.80%)
Dystonia	3/14 (21.43%)	6/45 (13.33%)	9/59 (15.25%)
Hypotonia	5/14 (35.71%)	29/45 (64.44%)	34/59 (57.63%)
Dysphagia	5/14 (35.71%)	11/45 (24.44%)	16/59 (27.12%)
Dysarthria	4/14 (28.57%)	12/45 (26.67%)	16/59 (27.12%)
Nystagmus*	1/14 (7.14%)	16/45 (35.56%)	17/59 (28.81%)
External ophthalmoplegia	3/14 (21.43%)	22/45 (48.89%)	25/59 (42.37%)
Ptosis	0/14 (0.00%)	8/45 (17.78%)	8/59 (13.56%)
**Involvement of other systems**			
Skin manifestation**	9/14 (64.28%)	8/45 (17.78%)	17/59 (28.81%)
Haematocytopenia**	9/14 (64.29%)	0/45 (0.00%)	9/59 (15.25%)
Repeated vomiting**	4/14 (28.57%)	0/45 (0.00%)	4/59 (6.78%)
Abnormalities in vision and hearing	4/14 (28.57%)	3/45 (6.67%)	7/59 (11.86%)
Cardiac involvement	3/14 (21.43%)	5/45 (11.11%)	8/59 (13.56%)
Abnormalities in vision and hearing*	4/14 (28.57%)	2/45 (4.44%)	7/59 (10.17%)
Skeletal deform	0/14 (0.00%)	2/45 (4.44%)	2/59 (3.39%)
**MRI**			
Basal ganglia	4/11 (36.36%)	11/43 (25.58%)	15/54 (27.78%)
Brainstem	1/11 (9.09%)	11/43 (25.58%)	12/54 (22.22%)
White matter signal abnormalities	4/11 (36.36%)	13/43 (30.23%)	17/54 (31.48%)
Cerebellum changes	2/11 (18.18%)	20/43 (46.51%)	22/54 (40.74%)
Cerebral atrophy**	9/11 (81.81%)	18/43 (41.86%)	27/54 (50.00%)
Thin corpus callosum	2/11 (18.18%)	1/43 (2.33%)	3/54 (5.56%)
Spinal cord	1/11 (9.09%)	10/43 (23.26%)	11/54 (20.37%)
**Outcome of treatment with Niacin/nicotinamide**			
Alive	2/4 (50.00%)	15/17 (88.24%)	17/21 (80.95%)
Deceased	2/4 (50.00%)	2/17 (11.76%)	4/21 (19.05%)

The asterisk represents statistical differences between patients with NAXD deficiency and NAXE deficiency, where * denotes *P* < 0.05 and ** denotes *P* < 0.01

In addition to neurological manifestations, patients with NAXD deficiency showed a significant tendency for involvement of multiple systems. The study found that approximately 27.12% (16/59) of patients developed skin symptoms. The incidence of skin lesions in patients with NAXD deficiency was as high as 57.14% (8/14), which was significantly higher than that in patients with NAXE deficiency (17.78%, 8/45). In addition, haematocytopenia and repeated vomiting were only observed in patients with NAXD deficiency, whereas skeletal deformities were only found in patients with NAXE deficiency (Table 1).

Regarding auxiliary examinations, patients with NAXD deficiency had a higher tendency to exhibit elevated serum lactate levels (35.71%) than those with NAXE deficiency. In contrast, patients with NAXE deficiency were more susceptible to increased cerebrospinal fluid lactate levels, accounting for 41.67%. In terms of imaging manifestations, multi-site involvement was observed in both types of patients. Among them, patients with NAXD deficiency are mainly affected in the basal ganglia and subcortical white matter and are more prone to cerebral atrophy during the late course of the disease. In contrast, patients with NAXE deficiency commonly exhibited lesions in the cerebellum, brainstem, and spinal cord (Table 1).

### Prognosis and treatment of patients with NAD(P)HX deficiency

The mortality rate of diseases associated with the NAD(P)HX metabolic repair system was as high as 66.67% (38/57), among which the mortality rates of patients with NAXD deficiency and NAXE deficiency were 85.71% (12/14) and 60.47% (26/43), respectively. For the 12 deceased patients with NAXD deficiency, the median age at death was 3 years (range: 0.33–32 years), and the median duration from symptom onset to death was 0.92 years (range: 0.08–4.5 years). Causes of death included respiratory failure (four cases), heart failure (one case), metabolic encephalopathy (one case), and severe infection (one case), with five cases having undetermined causes. Among the 12 deceased patients, only two received niacin/nicotinamide treatment during their lifetime, while the remaining 10 did not receive any treatment before death. One of the treated cases was a 4-month-old female infant who received low-dose niacin (50 mg/day) during the first month of the disease course [15]; her symptoms initially improved, and she was discharged after the first round of treatment; however, she died at 13 months of age due to the rapid deterioration of neurological symptoms caused by recurrent febrile infections. The second patient was a 32-year-old male patient who developed severe metabolic encephalopathy following trauma. Despite immediate initiation of a vitamin B_3_ treatment regimen (500 mg/day) upon diagnosis [16], the patient died of severe metabolic encephalopathy several months after onset due to irreversible progression of the condition.

For the 26 deceased patients with NAXE deficiency, the median age at death was 2.17 years (range: 0.5–22 years), and the median disease duration was 1.25 years (range: 0.08–4.0 years). Causes of death included respiratory failure (10 cases), heart failure (three cases), status epilepticus (two cases), metabolic encephalopathy (one case), and multiple organ failure (one case), with nine cases having undetermined causes. Among the 26 deceased patients, only two received niacin/nicotinamide treatment during their lifetime; one patient was a 2-year-and-10-month-old girl who presented with ataxia at onset, experienced rapid disease progression leading to respiratory failure and cerebral herniation on day 45 after onset, and died despite the initiation of intravenous 100 mg nicotinamide treatment on day 57 after onset [17]. The other patient was a 14-month-old boy who received the highest recorded dose of niacin (600 mg/day), with each dose increment demonstrating obvious transient improvement in the encephalopathic state and neurological symptoms, despite the condition deteriorating shortly afterwards, where signs of such transient improvement were clearly evident [18].

Among 57 patients reported in the literature and included in this study, 19 survived (33.3%), two (14.3%) had NAXD deficiency, and 17 (39.5%) had NAXE deficiency. Seventeen of these survivors received niacin/nicotinamide treatment (30–500 mg/day), whereas two NAXE-deficient patients survived without treatment, but with severe neurological sequelae [8, 38].

### Survival analysis in NAD(P)HX deficiency

**Fig. 4:**
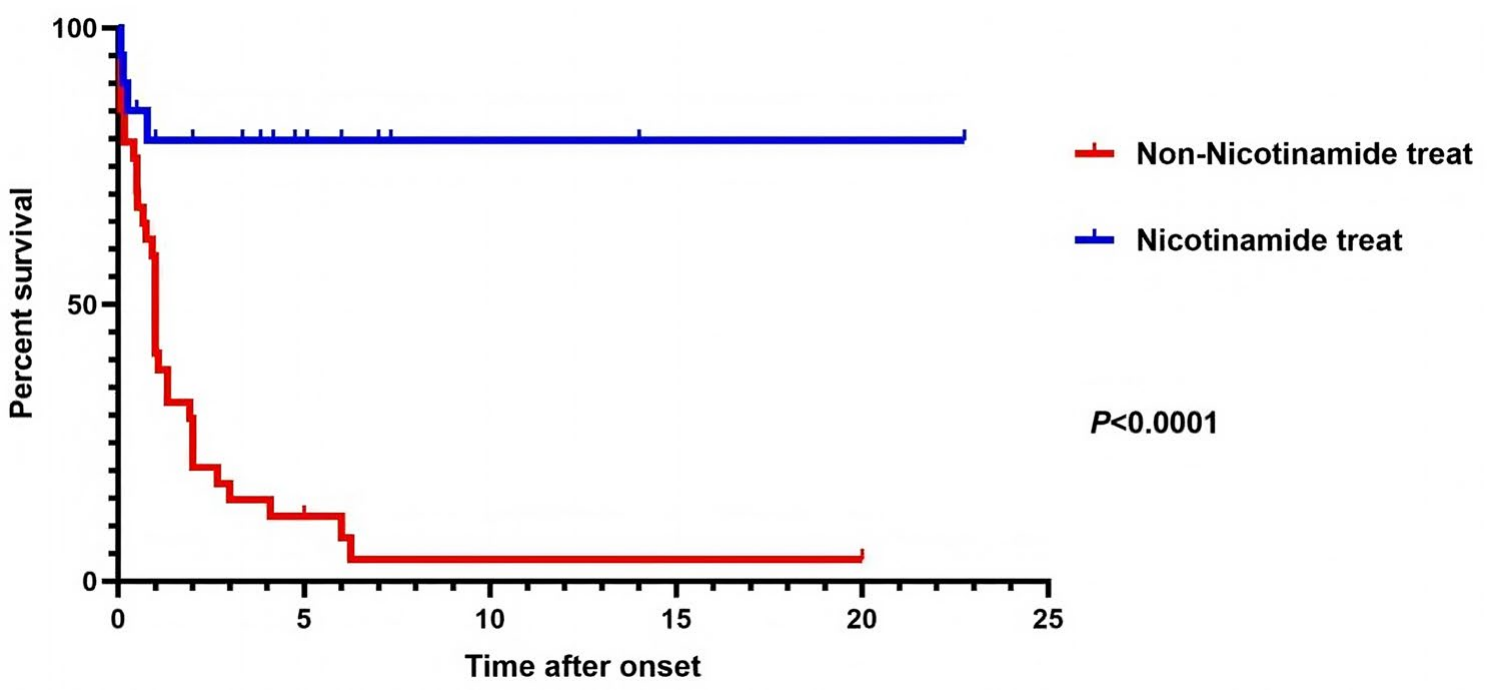
The survival curves of patients with NAD(P)HX metabolic deficiency who received niacin/nicotinamide treatment and those who did not receive nicotinamide treatment

We included 47 patients with complete case data to analyse risk factors for death. Univariate and multivariate Cox proportional hazards regression models confirmed that niacin/nicotinamide treatment was an independent protective factor for patient survival (HR = 33.43, 95% [CI] 6.42–154.23, *P* < 0.001) (Supplementary Table S2). Further analysis using a Kaplan–Meier survival curve for 54 patients with clear outcomes showed that the survival rate of patients who did not receive niacin/nicotinamide intervention declined precipitously within 2 years after disease onset. Among the 33 patients in the untreated group, 28 (84.85%) died within 2 years of disease onset, with a median survival time of only 1 year. Comparative analysis of survival curves revealed a significant difference in survival outcomes between the nicotinamide-treated and untreated groups (*P* < 0.0001, Fig. 4). The power analysis of the effect of nicotinamide treatment on mortality is detailed in the Supplementary Note.

### Genetic findings of patients with NAD(P)HX deficiency

**Fig. 5:**
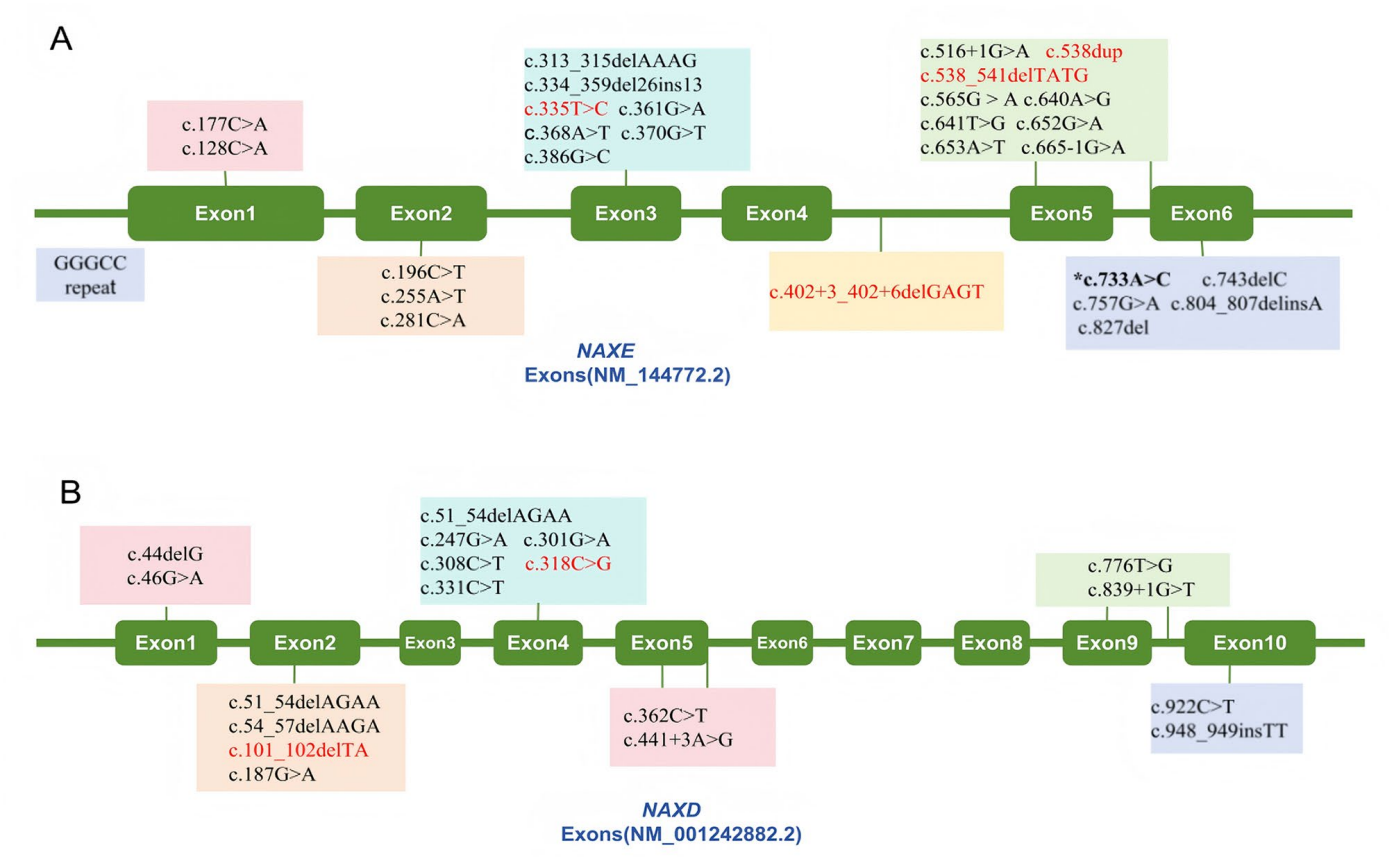
Variant sites and locations of the NAXE/NAXD gene reported in this study cohort and previous literature (**A**-**B**). The variant sites not reported in the literature are all marked in red font. *Common mutations

Twenty-eight distinct variants were identified in 45 patients with NAXE deficiency (Fig. 5A). No universal hotspot variants were found, but significant clustering was observed in the Chinese population: the c.733 A > C (p.Lys245Gln) variant in the NAXE gene accounted for 85.7% (12/14) of cases, nine of which were homozygous variants in the context of non-consanguineous marriages. Among the 31 non-Chinese patients, only one case each was identified in South Korea, Saudi Arabia, and South Africa carrying this variant. This variant was not detected in nine European patients, and there are no reports of NAXE gene mutations in the Americas to date. Additionally, 18 different variants were identified in 14 patients with NAXD deficiency (Fig. 5B), and no hotspot variants have been identified.

We further performed a comparative analysis of the general condition, clinical characteristics, imaging findings, and prognosis of 15 children carrying the c.733 A > C (p.Lys245Gln) variant and 30 children without this variant. There were no statistically significant differences between the two groups (Supplementary Table S3).

## Discussion

Herein, we describe the clinical characteristics, effects of nicotinamide, and prognosis of nine patients with NAD(P)HX metabolic deficiency caused by NAXE/NAXD variants admitted to our centre. By integrating the case cohort of this study with data from 50 cases reported in the literature, we found that nicotinamide treatment significantly improved the clinical outcomes of patients with NAD(P)HX metabolic deficiency, providing a basis for subsequent clinical treatment.

NAD(P)HX metabolic deficiency primarily manifests during infancy, although sporadic adult-onset cases have been reported. Involvement of the central nervous system is a typical clinical feature, with most affected patients exhibiting normal growth and development prior to onset but rapidly developing acute encephalopathy symptoms after onset, including developmental regression, ataxia, epilepsy, muscle weakness, and even coma or death due to brain herniation. In the early stages of the disease, ataxia, muscle weakness, epilepsy, or developmental regression often serve as the initial symptoms. In addition to central nervous system (CNS) manifestations, NAXD deficiency is likely to involve extracerebral organ systems such as the skin, haematological, gastrointestinal, and audiovisual system. The mortality rate of patients with NAXD deficiency (85.7%) was significantly higher than that of patients with NAXE deficiency (60.5%). In terms of response to nicotinamide treatment, the survival rate of the former was also lower than that of the latter. We speculate that this difference is closely related to the heterogeneity of the underlying metabolic mechanisms. As shown in Fig. 1B, when NAXD gene function is absent, toxic metabolites cannot be normally metabolised and continue to accumulate in the body, eventually leading to depletion of the NAD(P)H coenzyme pool [41]. In contrast, if only an NAXE deficiency exists, NAXD can convert S-NAD(P)HX metabolites into NAD(P)H. This hypothesis may explain the mechanism by which NAXD deficiency leads to more severe disease; however, further experimental data are required for verification.

One of the typical clinical features of this disease is that fever can induce symptom onset or prompt acute deterioration of the condition, with severe cases potentially progressing rapidly to respiratory failure, accounting for 81% of cases. Our study provides mechanistic support for this phenomenon: the NADH/NAD^+^ ratio in patients is significantly higher than that in normal individuals, with a more pronounced difference under 42 °C heat stress (Supplementary Fig. S1). Additionally, cyclic-NADHX levels progressively increase in NAXE-deficient cells under heat stress [9]. Therefore, avoiding infections and strictly controlling body temperature are crucial to prevent the onset of neurological symptoms, forming an important part of the treatment and management of the disease, which requires a strong clinical emphasis on timely vaccination, avoiding febrile diseases, and minimising triggering factors.

Characteristic cutaneous lesions are the second most common clinical feature of this disease. In the reported cases, although skin lesions were observed in only 28.8% of the patients, these lesions exhibited high clinical recognizability. Once specific skin lesions appear (especially in association with fever), they can serve as key clues for early diagnosis, indicating the need for the early initiation of nicotinamide intervention. Skin involvement predominantly affects flexural areas such as the neck, axillae, groin, and genitalia, with scattered lesions on the hands, feet, face, and trunk observed in a few patients (those with NAXE deficiency) [1]. The rash is characterized by well-demarcated erythema or changes resembling necrotizing epidermolysis, which can gradually progress to pathological changes, such as blister formation, epidermal necrosis, and desquamation. The rate of skin involvement in patients with NAXD deficiency (62.3%) was significantly higher than that in patients with NAXE deficiency (17.8%). Studies by Van Bergen et al. [41]. on the mechanism of skin damage showed that skin lesions only occurred in patients with concurrent cytosolic and mitochondrial NAXD deficiencies, while no skin involvement was observed in patients with isolated mitochondrial NAXD deficiency. This suggests that intact cytosolic NAXD isoforms exert a protective effect on the skin by maintaining the cytosolic NAD(P)H pool. The specific mechanism underlying skin damage in patients still warrants further investigation.

During the early stages of the disease, cranial MRI usually reveals no obvious abnormalities. However, as the disease progresses, subsequent MRI findings present diversity, even with variants at the same genetic locus. The affected areas of different patients not only vary but also show dynamic changes in lesion distribution; the initial involvement may be dominated by the cerebellum, which gradually progresses to basal ganglia or brainstem lesions. Commonly affected sites include abnormal signals in the basal ganglia and brainstem, cortical and subcortical white matter lesions, and cerebellar damage. In the acute phase, some patients may develop cerebral edema, which gradually progresses to cerebral and/or cerebellar atrophy as the disease progresses (particularly in patients with NAXD deficiency). Additionally, the spinal cord may have been involved in some cases (20.4%). Owing to significant fluctuations in the disease course, characterized by alternating remissions and exacerbations, patients often present with fever accompanied by acute neurological deterioration at onset, making them highly susceptible to misdiagnosis as encephalitis, meningitis, or autoimmune-mediated inflammatory diseases [9]. Routine and biochemical examinations of the CSF in this disease are mostly normal; however, CSF lactate is elevated in 37.2% of the patients, which provides an important clue for clinical differentiation. In this study, one NAXD-deficient patient (Pt-9) and one NAXE-deficient patient (Pt-3) were initially misdiagnosed with autoimmune encephalitis and acute cerebellitis, respectively, but were not definitively diagnosed through retrospective data analysis until years later. Additionally, in another two NAXE patients (Pt-6 and Pt-8), despite a persistent high clinical suspicion for mitochondrial disease, a definitive genetic diagnosis was not obtained during their lifetimes. Meanwhile, due to the lack of specificity in early clinical manifestations and cranial MRI, this disease is also easily confused with other neurometabolic diseases such as glutaric aciduria and methylmalonic aciduria [40]. Therefore, for patients with recurrent ataxia, abnormalities in muscle strength and tone, recurrent respiratory failure, and CNS involvement with or without skin manifestations, this disease should be suspected, and genetic testing should be initiated as early as possible.

NAD(P)HX metabolic deficiency is a lethal but treatable disease. Theoretically, NAXE/NAXD deficiency inflicts bodily damage primarily through two mechanisms: first, the depletion of nicotinamide adenine dinucleotide (NAD^+^), and second, the accumulation of toxic downstream metabolites that fail to be recycled back to NAD^+^—though the specific proportion of each mechanism’s contribution to the pathophysiological process remains elusive. However, this study clearly demonstrates that nicotinamide therapy is the sole key factor influencing patients’ survival rate, suggesting that the accumulation of toxic metabolites may exert minimal impact on disease mortality and that some scavenging or clearance mechanism may become operational once NAD^+^ levels are stabilized. Data indicate that 21 patients worldwide have received niacin/nicotinamide treatment, including four patients with NAXD deficiency (two survived) and 17 patients with NAXE deficiency (15 survived), with an overall survival rate of 80.95%, which is significantly higher than the 6.06% survival rate of untreated patients. The mechanism of nicotinamide treatment lies in the fact that studies have shown a significant depletion of nicotinamide adenine dinucleotide/phosphate (NAD(P)) cofactor reserves in patients with NAXE/NAXD deficiency. As a key precursor for the *de novo* synthesis of NAD^+^, nicotinamide can bypass enzymatic reaction disorders caused by NAXE/NAXD gene mutations. Restoring NAD^+^ levels through nicotinamide supplementation helps maintain normal cellular functions, ensures mitochondrial energy metabolism, and effectively alleviates disease symptoms while curbing disease progression [18, 42].

There is currently no unified standard for the treatment dosage of niacin/nicotinamide, with a wide range reported in the literature (30–600 mg/day), which provides a new research direction for the formulation of standardized treatment protocols in the future. We used high-dose nicotinamide treatment for seven patients with NAXE/NAXD deficiency and achieved good efficacy; all seven children survived, and three of them could attend school normally. Although Al-Amrani et al. [18]. proposed that excessive NAD(P)H might increase the accumulation of toxic metabolites in the body and aggravate the disease, this has not yet been confirmed. Our study showed that a dose of 500 mg/day is safe and effective for the Chinese population. In terms of tolerance, although no drug-related organ damage was observed in any of the children, skin rashes occurred in two cases during treatment. This suggests that determining the drug dosage with both optimal tolerance and efficacy remains an important direction for future studies. In addition, whether the derivatives of niacin (such as Nicotinamide Riboside and Nicotinamide Mononucleotide) can be used as potential therapeutic options is worthy of further exploration.

Therefore, in current clinical practice, nicotinamide therapy should be initiated as early as possible once NAXE and NAXD deficiencies are confirmed to improve patient outcomes and save lives. Meanwhile, due to the disease’s rarity resulting in extremely low clinical awareness and its diagnosis mostly relying on whole-exome sequencing (WES), it may have relatively serious underreporting and misdiagnosis. Thus, there is an urgent need to call for its inclusion in neonatal screening programs. Developing neonatal screening for this disease is of great practical significance and is expected to provide critical scientific evidence for improving the future screening system for treatable neonatal inherited metabolic disorders.

## Conclusions

NAD(P)HX metabolic defects are a rare group of treatable mitochondrial disorders with extremely high mortality rates. These disorders are primarily characterized by an onset in infancy, fever-triggered neurodegeneration, fluctuating early symptoms, and characteristic skin lesions in some patients. Early identification and timely initiation of nicotinamide therapy are crucial for improving patient outcomes and quality of life.

## Supplementary Information

Below is the link to the electronic supplementary material.

Supplementary Material 1: Supplementary figure S1. Results of detection of NAD^+^, NADH levels, and NADH/NAD^+^ ratio in skin fibroblasts from an NAXD-deficient patient and his healthy parents under 37 °C and 42 °C (heat stress) conditions (A-C). The NADH/NAD^+^ ratio of the patient was higher than that of the control group, and this difference was more pronounced under 42 °C. * denotes *P* < 0.05 and ** denotes *P* < 0.01

Supplementary Material 2

Supplementary Material 3

## Data Availability

Upon reasonable request, the corresponding author can provide the data supporting the study’s findings.

## Abbreviations

GADPH: Glyceraldehyde-3-phosphate dehydrogenase
Complex I: Mitochondrial Complex I
PDHc: Pyruvate dehydrogenase
CNS: Central nervous system
NAD: Nicotinamide adenine dinucleotide
NAD(P)HX: Nicotinamide adenine dinucleotide (phosphate), hydrated form
