# Supplementary figures and images for: Deficiency of the NAD(P)HX metabolic repair system: a treatable mitochondrial disease
Source: Orphanet J Rare Dis. 2026 Jan 23;21:63. doi: 10.1186/s13023-026-04218-4 (PMC12910793; doi:10.1186/s13023-026-04218-4)

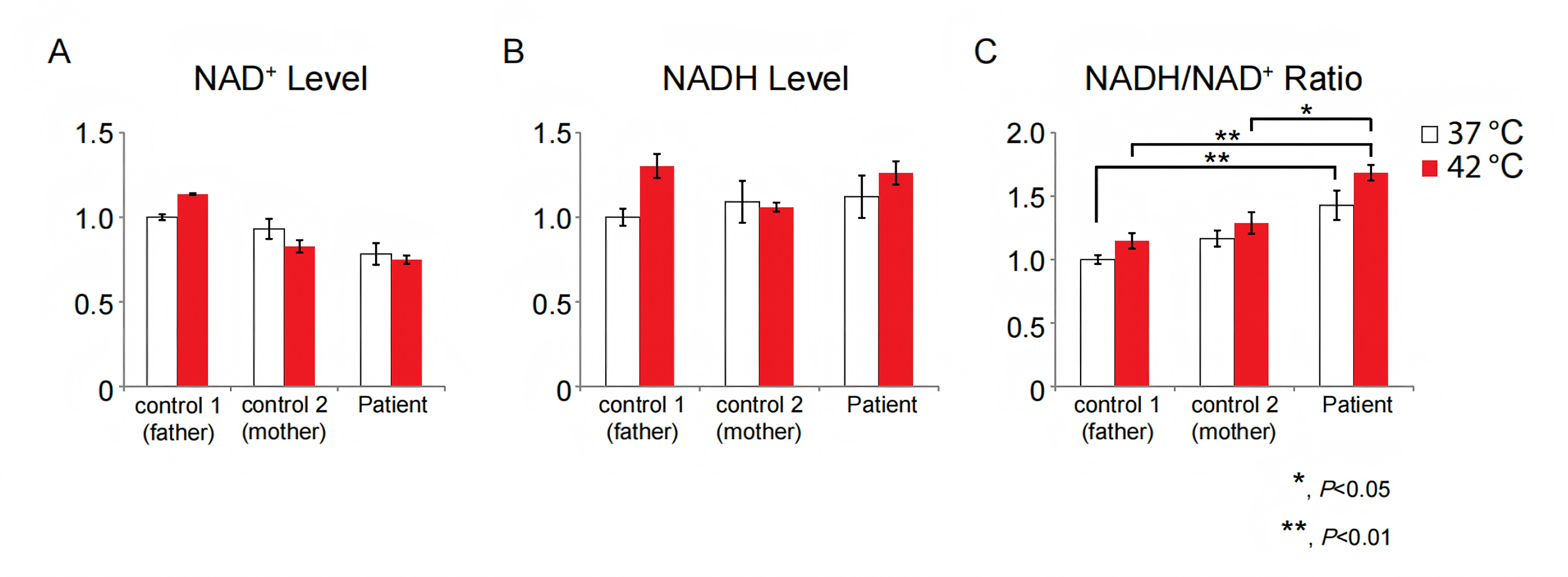

Supplement: Supplementary file 1 — Supplementary Material 1: Supplementary figure S1. Results of detection of NAD+, NADH levels, and NADH/NAD+ ratio in skin fibroblasts from an NAXD-deficient patient and his healthy parents under 37 °C and 42 °C (heat stress) conditions (A-C). The NADH/NAD+ ratio of the patient was higher than that of the control group, and this difference was more pronounced under 42 °C. * denotes P < 0.05 and ** denotes P < 0.01 [file 13023_2026_4218_MOESM1_ESM.png]
