# Supplementary material for: Deficiency of the NAD(P)HX metabolic repair system: a treatable mitochondrial disease
Source: Orphanet J Rare Dis. 2026 Jan 23;21:63. doi: 10.1186/s13023-026-04218-4 (PMC12910793; doi:10.1186/s13023-026-04218-4)
Supplement: Supplementary file 2 — Supplementary Material 2 [file 13023_2026_4218_MOESM2_ESM.docx]

**Supplementary Information**

**Deficiency of the NAD(P)HX metabolic repair system: A treatable mitochondrial disease**

Chaolong Xu^a,b^, Hong Jin^a^, Jiuwei Li^a^, Zhimei Liu^a^, Weihua Zhang^a^, Ji Zhou^a^, Ruoyu Duan^a^, Yang Liu^a^, Minhan Song^a^, Zixuan Zhang^a^, Tongyue Li^a^, Danmin Shen^a^, Ying Zou^a^, Junling Wang^a,c^, Hua Li^a^, Huafang Jiang^a,d#^, Fang Fang^a,b#^

^a^Department of Neurology, Beijing Children's Hospital, Capital Medical University, National Center for Children's Health, Beijing, China

^b^Laboratory for Clinical Medicine, Capital Medical University, Beijing, China

^c^Department of Pediatrics, The Third Affiliated Hospital of Zhengzhou University, Zhengzhou, China

^d^Department of Pediatrics, WeiFang Maternal and Child Health Hospital, Weifang, China

**Supplementary Table S1. Results of the *NAXE* genetic analysis of the 8 patients**

| Patient ID | Variationa | Originationb | Novel/reported(HGMD) | AFc | Predication of pathogenicity | | | | Fulfilled Criteria | ACMG |
| --- | --- | --- | --- | --- | --- | --- | --- | --- | --- | --- |
|  |  |  |  |  | Mutation taster | SIFT | CADD (score) | PROVEAN(score) |  |  |
| Pt-1 | c.733A>C(p. Lys245Gln) | M | Reported | 7.42·10^-5^ | Deleterious (1) | Deleterious(0) | 26.9 | Deleterious(-3.85) | PM2_Supporting+PM3_Strong+PP3 | LP |
|  | c.335T>C(p.Leu112Pro) | P | Novel | - | Deleterious (1) | Deleterious(0) | 32 | Deleterious(-7) | PM2_Supporting+PM3+PP3+PP4_Strong | LP |
| Pt-2 | c.538_541del(p.Tyr180Serfs*24) | P | Novel | - | - | - | - | - | PVS1+PM2_Supporting+PM3 | P |
|  | c.402+3_402+6delGAGT(p.？) | M | Novel | 2.83·10^-5^ | - | - | - | - | PM2_Supporting+PM3_Strong+PP3 | LP |
| Pt-3 | c.733A>C(p. Lys245Gln) | M | Reported | 7.42·10^-5^ | Deleterious(1) | Deleterious(0) | 26.9 | Deleterious(-3.85) | PM2_Supporting+PM3_Strong+PP3 | LP |
|  | c.733A>C(p. Lys245Gln) | P | Reported | 7.42·10^-5^ | Deleterious(1) | Deleterious(0) | 26.9 | Deleterious(-3.85) | PM2_Supporting+PM3_Strong+PP3 | LP |
| Pt-4 | c.733A>C(p. Lys245Gln) | P | Reported | 7.42·10^-5^ | Deleterious(1) | Deleterious(0) | 26.9 | Deleterious(-3.85) | PM2_Supporting+PM3_Strong+PP3 | LP |
|  | c.733A>C(p. Lys245Gln) | M | Reported | 7.42·10^-5^ | Deleterious(1) | Deleterious(0) | 26.9 | Deleterious(-3.85) | PM2_Supporting+PM3_Strong+PP3 | LP |
| Pt-5 | c.538dup(p.Tyr180Leufs*2) | M | Novel | - | - | - | - | - | PVS1+PM2_Supporting+PM3 | P |
|  | c.733A>C(p. Lys245Gln) | P | Reported | 7.42·10^-5^ | Deleterious(1) | Deleterious(0) | 26.9 | Deleterious(-3.85) | PM2_Supporting+PM3_Strong+PP3 | LP |
| Pt-6 | c.733A>C(p. Lys245Gln) | M | Reported | 7.42·10^-5^ | Deleterious(1) | Deleterious(0) | 26.9 | Deleterious(-3.85) | PM2_Supporting+PM3_Strong+PP3 | LP |
|  | c.733A>C(p. Lys245Gln) | P | Reported | 7.42·10^-5^ | Deleterious(1) | Deleterious(0) | 26.9 | Deleterious(-3.85) | PM2_Supporting+PM3_Strong+PP3 | LP |
| Pt-7 | c.733A>C(p. Lys245Gln) | M | Reported | 7.42·10^-5^ | Deleterious(1) | Deleterious(0) | 26.9 | Deleterious(-3.85) | PM2_Supporting+PM3_Strong+PP3 | LP |
|  | c.733A>C(p. Lys245Gln) | P | Reported | 7.42·10^-5^ | Deleterious(1) | Deleterious(0) | 26.9 | Deleterious(-3.85) | PM2_Supporting+PM3_Strong+PP3 | LP |
| Pt-8 | c.733A>C(p. Lys245Gln) | P | Reported | 7.42·10^-5^ | Deleterious(1) | Deleterious(0) | 26.9 | Deleterious(-3.85) | PM2_Supporting+PM3_Strong+PP3 | LP |
|  | c.733A>C(p. Lys245Gln) | M | Reported | 7.42·10^-5^ | Deleterious(1) | Deleterious(0) | 26.9 | Deleterious(-3.85) | PM2_Supporting+PM3_Strong+PP3 | LP |

^a^ The transcript used is NM_144772.2

^b^"P"is short for Paternal,"M"is short for Matermal

^c^The popilation frequendes are the global frequencies(ALL)of the gnomAD（v2.1.1）

The dash'-'denotes no records for the variant in the database,NA,not available

**Supplementary Table S2: Predictors of death among patients with NAD(P)HX deficiency**

| Risk factor | Univariate analysis | | |  | Multivariate analysis | | |
| --- | --- | --- | --- | --- | --- | --- | --- |
|  | *HR* | 95% CI | *P* value |  | *HR* | 95% CI | *P* value |
| Male | 0.74 | 0.24-2.24 | 0.77 |  | — | — | — |
| Age at onset (≤1years) | 0.39 | 0.19-1.30 | 0.15 |  | — | — | — |
| Normal development before onset | 0.88 | 0.21-4.16 | 0.98 |  | — | — | — |
| Epilepsy | 2.10 | 0.39-11.23 | 0.47 |  | — | — | — |
| Ataxia | 1.44 | 0.42-4.89 | 0.76 |  | — | — | — |
| Encephalopathy | 2.10 | 0.39-11.23 | 0.47 |  | — | — | — |
| Hypotonia | 0.61 | 0.21-1.83 | 0.41 |  | — | — | — |
| External ophthalmoplegia | 2.46 | 0.81-7.51 | 0.16 |  | — | — | — |
| Symptoms outside the nervous system | 1.12 | 0.36-3.54 | 0.94 |  | — | — | — |
| Elevated lactate | 0.29 | 0.08-1.23 | 0.07 |  | 0.44 | 0.89-2.45 | 0.42 |
| Basal ganglia | 0.25 | 0.06-1.04 | 0.06 |  | 0.53 | 0.14-1.91 | 0.38 |
| Brainstem | 1.30 | 0.35- 4.93 | 0.74 |  | — | — | — |
| White matter signal abnormalities | 0.44 | 0.15-3.06 | 0.12 |  | — | — | — |
| Cerebellum changes | 2.639 | 0.76-9.15 | 0.14 |  | — | — | — |
| Spinal cord | 1.14 | 0.28-4.68 | 0.88 |  | — | — | — |
| Niacin/Nicotinamide treatment | 46.75 | 9.37-233.23 | **<0.001** |  | 33.43 | 6.42-154.44 | **<0.001** |

HR = hazard ratio, CI = confidence interval.

**Supplementary Table S3: Comparative analysis of clinical characteristics and prognosis between carriers of c.733A>C (p.Lys245Gln) variant and non-carriers**

|  | c.733A>C (N=15) | Non-c.733A>C (N=30) |
| --- | --- | --- |
| **Age of onset (years)** | 1.84(0.83–3.17) | 1.33(0.00–22.00) |
| **Gender** |  |  |
| Female | 8/15(53.33%) | 12/30(40.00%) |
| Male | 7/15 (46.67%) | 18/30(60.00%) |
| **Symptoms of onset** |  |  |
| Developmental delay/regression | 3/15(20.00%) | 7/30(23.23%) |
| Ataxia | 11/15(73.33%) | 13/30(43.33%) |
| Epilepsy | 2/15(13.33%) | 2/30(6.67%) |
| Muscle weakness | 3/15(20.00%) | 8/30(26.27%) |
| Nystagmus | 0/15(0.00%) | 3/30(10.00%) |
| Others | 1/15(6.67%) | 14/30(46.67%) |
| **Main neurological symptoms** |  |  |
| Epilepsy | 8/15(53.33%) | 12/30(40.00%) |
| Ataxia | 12/15(80.00%) | 23/30(76.67%) |
| Encephalopathy | 11/15(73.33%) | 19/30(63.33%) |
| Dystonia | 2/15(13.33%) | 4/30(13.33%) |
| Hypotonia | 8/15(53.33%) | 21/30(70.00%) |
| Dysphagia | 6/15(40.00%) | 5/30(16.67%) |
| Dysarthria | 5/15(33.33%) | 7/30(23.33%) |
| Nystagmus | 5/15(33.33%) | 11/30(36.67%) |
| External ophthalmoplegia | 8/15(53.53%) | 14/30(46.67%) |
| Ptosis | 3/15(20.00%) | 5/30(16.67%) |
| **Involvement of other systems** | 3/15(20.00%) | 5/30(16.67%) |
| **Laboratory test** |  |  |
| Increased serum lactate | 3/12(25.00%) | 7/27(25.93%) |
| Increased cerebrospinal fluid lactate | 3/11(27.27%) | 12/25(48.00%) |
| **MRI** |  |  |
| Basal ganglia | 3/13(23.08%) | 8/30(26.67%) |
| Brainstem | 4/13(30.77%) | 7/30(23.23%) |
| White matter signal abnormalities | 3/13(23.08%) | 10/30(33.33%) |
| Cerebellum changes | 6/13(46.15%) | 14/30(46.67%) |
| Cerebral atrophy | 6/13(46.15%) | 12/30(40.00%) |
| Spinal cord | 3/13(9.09%) | 7/30(23.26%) |
| **Outcome of patient** |  |  |
| Alive | 9/15(14.29%) | 8/28(39.53%) |
| Deceased | 6/15(85.71%) | 20/28(60.47%) |
| **Outcome of treatment with Niacin/nicotinamide** |  |  |
| Alive | 8/9(88.89%) | 7/8(87.50%) |
| Deceased | 1/9(11.11%) | 1/8(12.50%) |
