# Supplementary material for: Deficiency of the NAD(P)HX metabolic repair system: a treatable mitochondrial disease
Source: Orphanet J Rare Dis. 2026 Jan 23;21:63. doi: 10.1186/s13023-026-04218-4 (PMC12910793; doi:10.1186/s13023-026-04218-4)
Supplement: Supplementary file 3 — Supplementary Material 3 [file 13023_2026_4218_MOESM3_ESM.docx]

**Supplementary Note 1**

**Power Analysis: Evaluation of Nicotinamide Therapy on Mortality Among All Cases**

A post-hoc power analysis was conducted to assess the ability of the study sample to detect the association between nicotinamide therapy and mortality, using G*Power 3.1 software.

**Study Data**

The analysis was based on 2×2 contingency table data of all cases:

Nicotinamide treatment group (n=21): 4 deaths, 17 survivors (mortality rate: 19.0%)

Non-treatment group (n=36): 34 deaths, 2 survivors (mortality rate: 94.4%)

Total sample size (N)=57

**Analysis Parameters and Results**

Statistical test: Chi-square test

Effect size: Cohen’s w=0.78 (calculated from observed and expected frequencies, indicating a large effect size per Cohen’s criteria: w≥0.5=large)

Significance level (α): 0.05 (two-tailed)

Statistical power (1−β): 99.6%

Supplementary Note 1
